# Meetings of Societies

**Published:** 1900-06

**Authors:** 


### MEETINGS OF SOCIETIES.

### Bristol Medico-Chirurgical Society.

*March 14th, 1900.*

Mr. W. H. Harsant, President, in the Chair.

Dr. F. H. Edgeworth showed a case of acromegaly. The patient, a female, had the usual enlargement of the hands and feet, mainly in the transverse diameter. A radiograph of the hand was shown. The enlargement was chiefly of the soft parts, the bones being but slightly increased in size. She had come complaining of numbness of her hands and arms. This made it difficult for her to dress. The symptoms had improved under treatment with tabloids of thyroid and pituitary gland substance.

Dr. J. Michell Clarke read a paper on "Two Cases of Aneurysm of the Thoracic, and one of the Abdominal, Aorta, with specimens," and showed a patient with thoracic aneurysm. Case I. was that of a porter. He gave no history of syphilis. A few days before coming to hospital he had been suddenly seized with dyspnœa and palpitation when going up a hill. There was œdema of the feet. The pulse was 100, but not that of aortic regurgitation. The apex beat was two inches outside the nipple. The cardiac dulness extended one inch to the right of the sternum, and upwards to the first intercostal space on the left side of that bone. There was a remarkably distinct thrill to be felt; it was continuous and extended into the carotid and subclavian areas. A loud murmur was heard best in the third left space, and conducted down the sternum. It could be heard at a distance of eight inches from the chest. The liver was enlarged, and showed true pulsation. As the weakness of the heart increased the thrill and murmur became less distinct, and the right ventricle dilated. There was some degree of cyanosis during the last three days of life. The urine had a specific gravity of 1022, and was very scanty. Anasarca increased towards the end, and ascites and pleuritic effusions developed. The *post mortem* showed a sacculated aneurysm of the root of the aorta on the right side. The wall of the aneurysm, which was thin, had perforated the pulmonary artery just above a cusp of the valve. The pulmonary artery was small, the conus arteriosus enlarged. Both ventricles were hypertrophied. Dr. Clarke remarked that varicose-aneurysm of the aorta had been diagnosed from the first. The difficulty had been in deciding where the perforation was. Communications with the *vena cava* were generally developed suddenly and associated with great dyspnœa. The position of the murmur and thrill, and the dulness to the left of the sternum, pointed to a communication with the pulmonary artery. Walshe says the communication is slowly established in some cases. Sir William Gairdner had published a case very similar to the speaker's. In both Gairdner's and his own cases there had been a striking degree of mental complacency in the patients. Case II.—This patient had felt quite well until three days before admission to the hospital. Then he had suddenly felt a tickling in the throat, and coughed up some blood. At the time he was quietly resting. This was twice repeated. He had pain in the left shoulder for some weeks previously to admission, and for some hours a pain over the heart. On admission, his sputum was bright red, viscid, and frothy. There was dulness behind the manubrium, and extending thence over the left apex. At this apex there was an absence of breath sounds, but with respiratory movement a rough sound like that of sawing wood was heard there. Also the second heart sound was accentuated. It was concluded that an aneurysm of the transverse arch of the aorta had by pressure caused collapse of the left apex of the lung. The patient died suddenly, coughing up a little blood. *Post mortem*, a large secondary cavity, bounded by connective tissue and fibrin, was found to have been excavated in the lung tissue. Case III. was one of abdominal aneurysm. The patient, a man, complained of paroxysmal pain in the right side of the abdomen. Often the attacks were of agonizing intensity, and in them the patient lay coiled up on the affected side with his hands over the painful region. There was tenderness at McBurney's point. Sometimes the pain passed to the testicle. The abdominal wall was very rigid. Slight hæmaturia sometimes followed the attacks. At the *post mortem* an aneurysm of the abdominal aorta was found to have eroded the vertebræ from the twelfth dorsal to the second lumbar, and there had been free extravasation of blood behind the colon. Dr. Clarke remarked on the difficulty in the diagnosis in this case. Iodide of potassium had relieved the symptoms for a time.—Dr. Waldo considered that interstitial nephritis was an important factor in the causation of an aneurysm, as Dr. West had recently pointed out, as well as syphilis, and certain kinds of work.—Dr. Groves described a case of aneurysm he had treated in which there had been continuous pain in the back for eighteen months. There had been dulness to the right of the sternum, and also at the left base below the angle of the scapula. Dyspnœa became a marked symptom. *Post mortem*, the arch of the aorta was found to be the size of a closed fist and lined with firm clot. A thin-walled portion of the sac pressed on the trachea. In the descending aorta was a second aneurysm, which had eroded the vertebræ extensively and ruptured into the pleura. Another case was mentioned in which a man had similar pain in the left side of the chest for a long period, the pain having first started when the man was at work. Damages had been claimed for this reason. The speaker regarded the case as one of aneurysm.—Dr. Watson Williams drew attention to the absence of paralysis of the vocal cord in Dr. Clarke's first case. He thought that it would be almost impossible for an uncomplicated aneurysm to give such marked symptoms without producing also some paresis of the vocal cord. He alluded to a case he had seen which was supposed to be one of recurring laryngitis from exposure, but in which paralysis of the vocal cord led him to diagnose aneurysm even in the absence of a swelling or pulsation. The man had subsequently died from sudden hæmoptysis.—Dr. Cave asked if a bruit had been heard on auscultation over the lumbar or dorsal region of the spine, as this sign often clinched the diagnosis of aneurysm of the abdominal or thoracic aorta.—Dr. Stack alluded to the use of the X-ray screen in the diagnosis of aneurysm. The method had been successfully used in five or six cases at the Bristol Royal Infirmary. In one case the diagnosis was made with the aid of the screen when no ordinary signs of aneurysm were present.—Dr. Edgeworth mentioned that he had found the administration of nitroglycerine vastly increase pain in the left arm of a patient who died later from aneursym, and suggested the possibility of the drug helping in the diagnosis of doubtful cases.—Dr. Waldo alluded to the relief of the pain of aneurysm he had obtained by giving the same drug.—Dr. Markham Skerritt would have naturally expected that nitro-glycerine and the nitrites would relieve rather than aggravate the symptoms of aneurysm, for it could not possibly act directly on the sac wall, but only peripherally on the vessels. He also pointed out that the existence of a communication between the two sides of the heart did not necessarily lead to cyanosis, as the blood of the two sides might not mix. In a case of patency of the foramen ovale, it is the concomitant stenosis of the pulmonary artery which leads to cyanosis.—Dr. Michell Clarke, in reply, said his cases did not bear out the view that interstitial nephritis was a common cause of aneurysm, and other cases he had seen also failed to support that view. The clinical symptoms in his first case were out of all proportion to the size of the aneurysm, and were due to the communication with the pulmonary artery.

Dr. F. H. Edgeworth read "Notes on a New Method of Treating Asthma." The treatment he wished to refer to was that by the injection of anti-diphtheritic serum. The case of a man of 24, who had been asthmatic six years, was instanced. Ten injections of 10 c.c. of Roux's serum was said to have completely cured the patient. A letter the speaker had received from Dr. Aikman, of Guernsey, who had treated many cases in this way, was quoted. Dr. Aikman had injected 500 units each time, the three first being given at intervals of two days. Improvement usually began after three injections. Some patients who had been asthmatic nearly all their lives had been reported cured. Dr. Edgeworth had followed Dr. Aikman's method. No skin rashes had resulted. There were two great classes of asthma—the bronchitic and the non-bronchitic. The serum had no curative effect in the non-bronchitic cases. In the bronchitic it caused watery expectoration for a few weeks, then subsidence, acting apparently like alkalies, and its efficiency depended upon its power of curing the bronchitis which was present. He had used it in eleven cases. In three there was no bronchitis, and the serum had no curative effect. In the remaining eight, three were cured, three relieved, and two unaffected.—Dr. Watson Williams remarked that the serum had been recommended in ozœna and chronic rhinitis, and had produced temporary benefit. It was astonishing how many drugs relieved neuroses! Rhinologists claimed to have cured a great number of cases of asthma. The majority of cases relapsed again sooner or later. He had tried the serum in atrophic rhinitis, and had been much disappointed with the results. He felt very doubtful as to how long the asthma cases would remain cured.—Dr. Edgeworth replied that the serum certainly did cure the bronchitis. Of course, if the bronchitis recurred the asthma would.

Mr. J. Taylor read a paper on "The X-ray History of a Fracture." Lantern slides were shown, reproduced from skiagrams. A report of this case will appear in the next issue of the *Journal*.

Dr. C. A. Hayman read a paper on "The Causes and Treatment of Toothache." The two most common causes of toothache were stated to be: (*a*) Exposure of the pulp or nerve, and (*b*) dental abscess. In the first variety the dentine becomes affected with caries through the existence of a hole or flaw in the enamel. Through the aperture fluids enter the dentine and gradually permeate it. Softening of the dentine by acid fermentation ensues, and in course of time, when biting some hard substance, the enamel gives way. The softened dentine then becomes pressed upon the pulp of the tooth, causing great pain. In such a case the indications were to dry the cavity and relieve the pressure by removing all food *débris* and broken enamel. This should be done by working carefully with excavators round the edge of the cavity, avoiding the centre of the tooth. Thus the pressure and pain would be relieved. Usually when the cavity had been quite cleaned a small bleeding point would be seen, and that was the exposed pulp. The exposure, if small, should be capped with a small disc of writing-paper having a paste of iodoform, carbolic acid. and glycerine on the side facing the dentine. This will protect from pressure, and encourage the deposition of secondary dentine. Finally, the tooth can be filled in the usual way, and will then last for years. If the exposure is too large for capping, the pulp should be destroyed, the nerve canal disinfected thoroughly and filled with guttapercha points softened by chloroform, and then the cavity can be filled in the ordinary way. In the second variety, the abscess was caused by the death and decomposition of the nerve or pulp of the tooth. For a time the nerve channels may form a natural drain, but later they become blocked, and then the pus forces its way out at the apex of the fang and infects the surrounding tissues. Pain, heat, redness, and swelling of the face then ensue. In treating such cases the pulp chambers and nerve canals must be thoroughly opened and cleansed with nerve extractors and drills, then filled as described before. A gold cap can then be fixed on the tooth. By adopting such measures as the above, it was quite possible to save teeth though they were badly decayed and had even formed abscesses. If the dental periosteum of a tooth became detached, the pulp also having suppurated, it was past conservative treatment, and should be extracted. Practitioners were recommended to keep in their possession a few dental instruments as a first-aid equipment. The paper was illustrated by the aid of lantern slides, and instruments and sections of teeth were shown.

---

*April 11th, 1900.*

Mr. W. H. Harsant, President, in the Chair.

Dr. H. Waldo showed a male patient, aged 19, who had a large area of brownish pigmented skin in the left posterior hip region. The patient complained of loss of energy in mind and body. He thought the pigmentation had existed three or four years. For some weeks he had been troubled with indigestion and nausea, and also greatly with flatulence. Studying for an examination had aggravated his symptoms. The genitals were also discoloured in a similar way, but pigmentation had not been observed in the mouth. There was giddiness on first getting up in the morning. The heart-sounds were feeble, the pulse soft. Tabloids of supra-renal extract had been given and also diastol. Some improvement had resulted from the treatment. Dr. Waldo thought the case resembled one of Addison's disease more than anything. It was recognised that the pigmentation in that disease might appear years before other symptoms. Dr. Waldo also showed a case of urticaria pigmentosa. There was no pigmentation in the mouth. The disease had begun when the child was between three and four years of age in this case. Often, in others, it began in the first six months of life. It differed from ordinary urticaria in its permanency. The eruption might be elevated or not. It often continued to the age of puberty. Iodide of potasium had been found to relieve the itching in this case. In another case cold morning sponging had been found beneficial.—Dr. G. Parker thought, in the first case, that somethickening of the skin existed where the pigmentation was seen, and that the growth of hair was excessive, suggesting, in the absence of any history, that it was a birth-mark. He alluded to the association of pigmentation, dyspepsia, and flatulence which had been remarked upon by Rolleston.—Dr. WATSON WILLIAMS agreed with the diagnosis of Addison's disease, and thought all the classical symptoms should not be waited for before making a diagnosis. He alluded to a case he had seen in which there had been present vomiting and dicrotism of the pulse, followed later by slight pigmentation. That case had ended fatally. He thought some cases were not recognised because the pigmentation was slight in degree. He mentioned also the case of a boy of fourteen with urticaria pigmentosa he had seen. When the boy became heated the irritation increased.—Dr. WALDO replied that he attached as much importance to the pigmentation of the genitalia as to that in the hip region.

Dr. A. J. HARRISON showed two children affected with favus. The yellow crusts had disappeared after a few applications of boracic ointment. Very few spores were to be found in the hairs, but the scutula teemed with them.

Dr. WALTER SWAYNE showed a specimen of ovarian tumour, and read notes on two cases of hysterectomy for fibroids with intra-peritoneal treatment of the pedicle. (For the notes on the hysterectomy cases, see p. 123.) The ovarian tumour had been removed from a multiparous woman, aged 39. It had extended upwards to midway between the umbilicus and ensiform cartilage. The patient had a cough, swelling of the legs, dulness at the bases of the lungs, and seemed to be in an almost hopeless condition. Dark brown fluid was withdrawn from the cyst after exposure. The point of greatest interest was the extremely rotten state of the cyst wall. It would not hold forceps. Numerous adhesions had to be separated and the vermiform appendix to be removed. Many pieces of the tumour had to be separately removed from amidst the intestines. The pedicle had been secured by triple interlocking ligatures. A pint and a half of fluid was left in the abdomen to combat the shock. Recovery ensued, the patient being discharged within a month.—Dr. AUST LAWRENCE remarked on the difficulty of explaining the rottenness of the walls of the ovarian cyst which had been shown, as there were numerous adhesions, and the pedicle, which was thick, had not been twisted. Speaking of fibroids, he had seen nearly a thousand cases in twenty-five years, and in not more than 5 per cent. of these would radical surgical treatment have been justified; 40 per cent. of the remainder would have been rendered very comfortable by the help of minor surgery and medicine. Enucleation was dangerous, for the interference easily caused necrosis. Hospital patients required operations when the wealthy did not. When fibroids were small, vaginal hysterectomy would be the operation of choice; but it was not justifiable to operate in the early stage of the disease, except under special circumstances, because no one could tell if the disease would progress or not. The patient must be watched. Keith had given up operating on fibroids, and used Apostoli's electrical treatment. He knew of ten cases in which women with fibroids had become pregnant, and had had no trouble in parturition. Some women bore loss of blood much better than others. He had known women lose blood three weeks out of every four and yet remain well. Pressure symptoms in some cases might be relieved by the alteration of the position of the uterus.—Mr. ROGER WILLIAMS remarked that the Registrar-General's reports did not show what proportion of women in the general population had myoma, nor what proportion died from the disease. He estimated, from a consideration of the St. Bartholomew's Hospital *post-mortem* records and other data, that 25 per cent. of women over thirty-five had myomata. As there were about 4,000,000 women of such age in the country, this meant that about 1,000,000 women suffer from the affection. Taking the Registrar-General's returns under the heading of diseases of the uterus and vagina, and eliminating deaths from cancer and parturition, it appeared that about 500 women died from myoma per annum, and 500 out of 1,000,000 is equivalent to 1 in 2,000, and he believed that was the most correct statement available with regard to the mortality of the disease. These numbers indicated how rarely operation was likely to be called for in the affection. From ovarian tumours a much larger proportion of women died. —Dr. Newnham had been surprised not to hear that a twisted pedicle had been the cause of the rottenness of the cyst wall in Dr. Swayne's ovarian case. He had had recently two cases in which twisted pedicles had caused extensive adhesions.—The President thought that there was a tendency to operate on fibroids too frequently. Oöphorectomy would not be much more heard of as a remedy, and Apostoli's method would share the same fate. It seemed to him also that in the cases not amenable to operation by the vaginal route the abdominal operation with intra-peritoneal treatment of the pedicle would become the one most frequently selected.—Dr. Walter Swayne replied that he had no faith in Apostoli's treatment. It seemed to stop hemorrhage in some cases The size of the tumour alone did not afford any indication. as to the necessity of operation.

Dr. Newnham read notes on two successful cases of operation for imperforate hymen with retained menses. The first, that of a girl of 18, had been seen for supposed retention of urine. There was an abdominal tumour, and the hymen was tense, and blueish in colour. Thick treacly fluid was evacuated by incision. The second case was very similar, and had been treated in the same way. Both patients had uneventful recoveries.—Dr. James Swain mentioned a case of the kind he had seen in which the tumour reached nearly to the umbilicus, but in which there had been absolutely no vagina present. He had made a transverse incision and dissected upwards between the bladder and rectum. The bladder was wounded twice and the wounds sutured. The distended uterus was opened at a later date through the newly-formed vaginal canal after the bladder wounds had closed.—Dr. Aust Lawrence recollected having seen a case tapped through the rectum. The contents had putrefied in consequence. In another case, occlusion of the canal had followed sloughing of the cervix uteri. He had had also a case of double uterus in which on one side menstruation was normal, but on the other there was retention of the menses. The distended portion of the uterus had been tapped with a small trochar and washed out. This patient died from rupture of the Fallopian tube on the affected side.

Dr. Stack read a paper on a case of leontiasis ossia, and showed the skull and other bones he had obtained at the *post mortem* and photographs of the case. The patient, a female, was 21 years of age at the time of death. Her death had occurred suddenly from asphyxia. For ten years, off and on, the patient had been under observation at the Infirmary. Her mother had first noticed the size of the head becoming abnormal when the patient was seven years old. As the size of the head increased the patient used to support it against the wall or on the table. The pharynx had been pushed forward. The femora and tibiæ became bent, and all the bones were affected in some degree. Parts of the skull were nearly three inches thick; but the intra-cranial cavity was almost normal, and the brain and pituitary body were normal. There was a naso-pharyngeal sarcoma present containing myeloid cells. The deformity of the long bones and pelvis was due to the increase of the weight of the head. There was no definite evidence of rachitis nor of syphilis, though the latter could not be excluded.—Mr. Roger Williams remarked on the excellence of the specimens shown, and alluded to one of Murchison's he had seen. He considered leontiasis the best heading under which to place them in the present state of knowledge. The enlargement seemed to centre in the jaws at first, and the dacryops from which this patient had suffered suggested that the upper jaws had been early affected. Discussing the ætiology, he considered cases of this kind were possibly due to nerve lesions near the base of the brain, and that syphilis might be a factor in the causation.—Mr. Mackie asked if atheromatous changes in the blood-vessels had been found, as they had been conspicuous in a case published by Dr. Young.—Dr. Stack replied that the aorta and kidneys had been found healthy.

Mr. Ackland showed some new instruments he had devised for the treatment of antral suppuration. These included a fluted perforator, which easily cuts its way into the antrum through the alveolar border of the jaw; hollow gilded screws to be fixed in the canal made by the perforator, and provided with a cap, which could be easily removed, to prevent the entrance of food; a two-way nozzle to fit into the hollow screw, and which the patient can adjust himself, and with it syringe out the cavites; and an instrument for measuring the length of the canal leading into the antrum. With these instruments the perforation can be made, and the screw drainage-tube introduced under one administration of gas.—Dr. Watson Williams remarked that antral disease was commoner than is generally supposed. The majority of rhinologists believed in trying these less radical methods of treatment first. He thought Mr. Ackland's method likely to come into very general use.—Dr. James Swain said he had sometimes passed a drainage-tube in through the tooth socket and out through the nose. He considered Mr. Ackland's method a very excellent one.

J. Lacy Firth, *Reporter.*
G. Munro Smith, *Hon. Sec.*

---

*May 9th*, 1900.

Mr. W. H. Harsant, President, in the Chair.

The proceedings opened with the presentation of a testimonial to Mr. L. M. Griffiths. (See pp. 97—103.)

Mr. A. Carr showed a soldier invalided from South Africa, who was shot in both arm and chest at Paardeberg on February 18th.

Dr. Newnham exhibited a uterine myoma he had removed by the intra-peritoneal method.

Dr. Theodore Fisher read notes on some cases of vascular lesions in children. In four of these thrombosis of the cerebral veins was present; in two others there was intra-cerebral hemorrhage. The thrombosis occurred as a complication of acute diarrhœa, broncho-pneumonia, noma, and ascites from hepatic cirrhosis.

Dr. Edgeworth read a paper on some cases of Acroparæsthesia which had come under his care. They occurred in women between forty and fifty, and the chief symptoms were sensations of tingling and anæsthesia in the arms and hands with occasional cramp-like pains. There were no signs of vaso-motor disturbance. Nitro-glycerine and bromides were found useful.

Drs. Stack, Michell Clarke, and Cave remarked on the cases.

Mr. A. W. Prichard read a paper on three cases of abdominal surgery presenting somewhat unusual features. The first was a case of perforated gastric ulcer, where the perforation healed, although it could not be stitched up. In the second, intestinal obstruction followed old appendicitis, and was relieved by enterectomy. In the third (a solid ovarian tumour) intestinal obstruction followed the operation of removal from adhesion of the intestine to the abdominal wound. This was cured by operation.

J. Lacy Firth, *Reporter.*
Munro Smith, *Secretary pro tem.*

